# Preparation and characterization of active Cirish fructans–fish gelatin film: Physicochemical, antioxidant, and antimicrobial properties

**DOI:** 10.1002/fsn3.3106

**Published:** 2022-10-26

**Authors:** Morteza Jafari, Rana Afkhami, Nasser Sedaghat

**Affiliations:** ^1^ Department of Food Science and Technology Ferdowsi University of Mashhad Mashhad Iran

**Keywords:** Cirish (*Eremurus spectabilis*), DSC, fructans, FTIR, morphology

## Abstract

This study was aimed to evaluate the film‐forming ability and characterization of ultrasonically extracted Cirish fructans (CF) and CF–cold‐water fish gelatin (G) composite films. The film‐forming solutions were prepared at different levels (CF100‐0G, CF75‐G25, CF50‐G50, CF25‐G75, and CF0‐G100) and the corresponding data were analyzed based on one‐way analysis of variance. The results indicated that CF addition led to an impressive increase in composite films thickness (69.38–86.45 μm), moisture content (16.05%–27.8%), surface hydrophobicity, tensile strength (5.73–17.89 MPa), elongation at break (0.83%–1.66%), Young's modulus (77.12–88.15 MPa), and Tg (38.83–47.4°C) which CF75‐G25 had the highest values. Meanwhile, the solubility (77.12%–88.15%), WVP (1.89–2.86 × 10^−10^gm^−1^ s^−1^ Pa^−1^), and oxygen permeability (1.53–3.26 × 10^14^ cm^3^ m^−1^ s^−1^ Pa^−1^) of the composite films decreased. The FTIR spectra indicated the protein–polysaccharide interactions and revealed that the secondary structure of gelatin was shifted from triple‐helix structure (1661 cm^−1^) toward α‐helix structure (1650–1657 cm^−1^) when CF was incorporated. The microstructure observations indicated that unlike gelatin film, CF film exhibited the smooth and uniform surface without cracks and phase separation. It was found that CF films had high total phenolic content (6.73 mg GEA g^−1^) and showed DPPH radical scavenging activity (67.86%). On the other hand, it showed inherent antimicrobial activity against both gram‐positive and gram‐negative bacteria. The results indicated that CF and CF‐cold fish gelatin have great potential as active films with improved physical, mechanical, and barrier properties.

## Graphical abstract

The composite films were prepared by Cirish fructans and cold‐water fish gelatin. FTIR, DSC, and SEM analyses were demonstrated the good miscibility and compatibility of the two polymers. Addition of Cirish fructans improved physical, structural, and gas and water barrier properties of composite films and Cirish fructans incorporation presented composite films with antioxidant and antimicrobial activities.

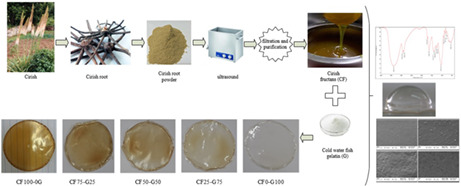

## 1 INTRODUCTION

In recent years, the demand for active films based on biopolymers instead of synthetic polymers has been of interest because of environmental and biodegradability properties (Chambi & Grosso, 2011; Hanani et al., 2012). Active packaging is gaining development due to its potential to prolong shelf life and preserve food quality and safety. Active food packaging is a new concept in which active compounds such as antioxidants and antimicrobial agents or compounds with inherent preservation capacity incorporate into packaging materials (Araghi et al., 2015; Ashrafi et al., 2018). Typically, polysaccharides, proteins, and lipids are the main compounds to prepare biopolymer‐based film. One of the most abundant, nontoxic, and popular biopolymers is gelatin. Gelatin has been applied extensively for protein‐based biodegradable films. It commercially manufactures from partial hydrolysis or thermal degradation of the collagen presented in bones and skins of animal and fish (Hanani et al., 2012). Structurally, gelatin has a triple‐helix structure stabilized mainly by the formation of interchain hydrogen bonds between carbonyl and amines groups (Dai et al., 2006; De Meutter & Goormaghtigh, 2021; Shi et al., 2022). It consists of nonpolar aliphatic amino acids, including glycine, proline, alanine, and hydroxyproline and has more linear structure compared with globular proteins (De Meutter & Goormaghtigh, 2021; Nuanmano et al., 2015; Payne & Veis, 1988; Shi et al., 2022).

Among different gelatin sources such as pig skin, cowhide, beef, camel bones, and fish, the fish gelatin which extracts from fish skin and bones residues is an inexpensive source for substitutes of bovine and porcine gelatin. The consumption of pork gelatin is limited because of immunological and ethno‐religious concerns and is considered unlawful in Judaism and Islam (González et al., 2011; Mahmood et al., 2022). On the other hand, the acceptance of bovine gelatin in Muslims and Jews depends on the method of preparation which should be according to religious dietary law. Additionally, bovine gelatin is forbidden by vegetarian and there is risk of bovine spongiform encephalopathy (González et al., 2011; Mahmood et al., 2022; Shi et al., 2022). Furthermore, Fish gelatin‐based film has been promoted recently duo to its myofibrillar protein content that enhance film manufacturing with good barrier to ultraviolet and oxygen (Nuanmano et al., 2015). However, fish gelatin has lower melting point and forms softer gel compared to mammals gelatin due to its low amount of proline and hydroxyproline content which are important for forming hydrogen bonds during film preparation (Dai et al., 2006). On the other hand, gelatin film is brittle and susceptible to crack due to the strong cohesive energy density of the polymer and has high water vapor permeability because of its hydrophilic character (Chambi & Grosso, 2011; Nuanmano et al., 2015). Therefore, mechanical and water resistance of fish gelatin‐based films should be improved.

Cirish (*Eremurus spectabilis*) is a plant that belongs to the family of *Liliaceae*. The roots of Eremurus species are rich of oligo‐ and polysaccharides, including branched arabinogalactan, linear galactomannan, and short chains of fructose units with a single d‐glucosyl unit at the nonreducing end that accumulate during their growth (Flamm et al., 2001; Karaman et al., 2011; Muhidinov et al., 2020; Pourfarzad et al., 2015; Smirnova et al., 2001). The term fructans refers to prebiotic components, including fructo‐oligosaccharides with average chain length of 2–9 units or oligo‐fructose with the longer chains (chain length ≥ 10) which are called inulins (Flamm et al., 2001; Muhidinov et al., 2020; Pourfarzad et al., 2015). Fructans are interesting for their techno‐functional characteristics, such as bulking agent, water retention, and fat substitute. Meanwhile, it was reported that Cirish root solution is a rich source of phenolic components with antioxidant and antimicrobial activity (Flamm et al., 2001; Kanaani & Mohamadi Sani, 2015; Karaman et al., 2011; Tuzcu et al., 2017).

Different synthetic antioxidants have been used previously to enlarge the shelf life of food products. However, their usage is limited because of toxicity. Therefore, the incorporation of plant extracts with antioxidant and antimicrobial activities have been focused to develop biological activity of the films (Gómez‐Estaca et al., 2009; Gómez‐Guillén et al., 2007). However, to the best of our knowledge, there is limited information regarding developing prebiotic biopolymer‐based films with inherent antioxidant and antibacterial properties at the same time. On the other hand, until now, there is no study regarding the preparation of CF film. Therefore, this study was aimed to (i) prepare CF‐based film with antioxidant and antibacterial properties and (ii) develop CF–cold fish gelatin film with superior mechanical and barrier properties. To this end, prepared films were characterized in terms of structural, morphological, mechanical, thermal, water vapor permeability, gas permeability, antioxidant, and antimicrobial properties.

## 2 MATERIAL AND METHODS

### 2.1 Raw materials

For this research, the Cirish root powder was purchased from the local medical plant market, Mashhad, Iran and then were passed through a 60‐m sieve screen and stored at 4°C for further use. Cold‐water fish gelatin was purchased from Sigma‐Aldrich.

### 2.2 Chemical characteristics of Cirish root powder

The chemical characterizations, including moisture, ash, fat, and total dietary fiber, were determined according to the standard AOAC methods (AOAC, 1995). The Kjeldahl method was used to measure the crude protein content and 6.25 was considered as the conversion rate of nitrogen to crude protein. All examinations were done at least two times.

### 2.3 Extraction of Cirish fructans by indirect sonication extraction

The fructans of Cirish root were extracted under indirect sonication using ultrasound cleaning bath (model Ultrasonix OS 280R, Schuder Schal GmbH & Co., KG) operating at a frequency of 25 kHz with input power of 500 W. Cirish root powder was diluted with solvent: solid ratio of 50:1 and exposed to sonication amplitude of 80%. The extraction period was 30 min at 60°C with periodically shaking. After that, the suspension was cooled until 35°C and filtered through muslin cloth to remove the insoluble residues followed by centrifuging at 4500 *g* for 15 min to collect the supernatant. Pourfarzad et al. (2015) have shown that the indirect sonication extraction is the most suitable method for fructans extraction among conventional extraction, direct sonication extraction, and indirect sonication extraction.

### 2.4 Properties of Cirish root solution

The properties of Cirish root solution, including total carbohydrate, reducing sugar, Fructans content (Extraction yield), Fructans purity, the extracted Fructans average chain length and average particle size, and the Zeta potential of Fructans, were evaluated (Lingyun et al., 2007; Pourfarzad et al., 2015).

### 2.5 Preparation of the films

The film‐forming solutions (5% w/v) were prepared using CF and cold‐water fish gelatin at different proportions (%) of 0:100, 50:50, 75:25, and 100:0, respectively. CF was maintained at 85°C for 30 min with magnetic stirring. Fish gelatin was dissolved in distilled water at 60°C for 30 min until a clear solution was obtained. The prepared solutions were mixed together at 60°C with stirring for 30 min, followed by addition of glycerol (25% w/w based on dry solid) with constant stirring for another 30 min. The mixture was then cooled to room temperature. The film solutions (70 g) were then cast onto petri dishes (15, 5 cm, and 3 mm) and dried at room temperature for 24 h. All films were peeled from the plates and stored in a desiccator containing saturated sodium bromide (NaBr) solution conditioned at 56% (RH) and 30°C before further analysis.

### 2.6 Film thickness

The thickness of each film in five locations was measured using a digital micrometer (Mitutoyo) with 0.001 mm of accuracy.

### 2.7 Moisture content

The moisture content of films (0.5 g) was measured gravimetrically at the end of the drying process (105°C in an oven under air circulation for an adequate time to achieve a constant mass).

### 2.8 Water solubility

In order to determining the water solubility (WS), the conditioned film samples (2 × 3 cm; dried in an oven at 105°C for 24 h) were weighed and immersed in 30 ml of distilled water at 50‐ml centrifuge tube followed by shaking at a speed of 250 rpm for 24 h at 25°C. Samples were centrifuged at 3024 *g* for 15 min. WS was calculated after drying of samples at 105°C for 24 h according to the following equation (Chambi & Grosso, 2011):

(1) $$\mathrm{WS}\;\left(\%\right)=\frac{\left(M_{i}\times \left(100-\mathrm{MC}\right)-M_{f}\right)}{M_{i}\times \left(100\;‐\;MC\right)}\times 100,$$

where *M*_*i*_ is the initial film weight (g). *M*_*f*_ is the final dry weight of film nonsolubilized in water (g). MC is the moisture content.

### 2.9 Water vapor permeability

The water vapor permeability (WVP) test was conducted gravimetrically according to ASTM Standard E96/E96 M‐16 (ASTM, 2016) with some modifications (Gómez‐Guillén et al., 2007).

### 2.10 Oxygen permeability

Oxygen permeability (OP) of films (50 cm^2^) was determined at 53% RH and 25°C using an Oxtran system (Mocon), following the standard method of ASTM (ASTM D3985‐17, 2017).

### 2.11 Surface hydrophobicity

Water contact angle was utilized to represent the surface hydrophobicity of the films. The contact angle measurements were carried out according to the sessile drop method on a goniometer (Krüss G10), equipped with an image analysis software. About 5 μl of ultrapure water was deposited on the film surface with a precision syringe. Five measurements per films were carried out at 25°C.

### 2.12 Scanning electron microscopy (SEM)

The microstructure of surfaces and cryo‐fractured cross‐sections of the films were observed using a scanning electron microscope (VP 1450; LEO Company). Samples were mounted on an aluminum stub with double‐sided stick tape and were coated with gold. The images were captured using an accelerating voltage of 20 kV with a magnification of 500×, 1000×, and 2000×.

### 2.13 Mechanical properties

The mechanical properties (tensile strength [TS], elongation at break [EAB], and Young's modulus [YM]) of prepared films (10 × 2 cm^2^ sizes) were performed following the ASTM‐D882‐18 standard (ASTM, 2018).

### 2.14 Thermal properties

Thermal properties of films (conditioned in a desiccator at 25°C and 0% RH for 10 days) were investigated using a Differential Scanning Calorimeter (DSC; DSC‐60; Shimadzu). Approximately 5 mg of each film was subjected into a double heating–cooling cycle from −50 to 150°C with a heating rate of 10°C min^−1^ under a nitrogen gas flow rate of 25 ml min^−1^. The empty aluminum pan was used as a reference. The second heating run was considered for the determination of the glass transition temperature (Tg).

### 2.15 Fourier transform infrared spectroscopy (FTIR)

The FTIR spectra of the films were measured at 25°C in the range of 400–4000 cm^−1^ with 32 consecutive scans at 8 cm^−1^ resolution using a PerkinElmer FTIR (Spectrum Two).

### 2.16 Determination of antioxidant properties

The total phenolic content (TPC) and the DPPH free radical scavenging activity of films were measured according to the Folin–Ciocalteu method (Gómez‐Estaca et al., 2009; Gómez‐Guillén et al., 2007) and the method of Kedare and Singh (2011), respectively.

### 2.17 Determination of the antimicrobial activity

The antimicrobial activity of the films against food pathogens, including *Escherichia coli* O157:H7 (G −), *Bacillus subtilis* (G +), *Listeria monocytogenes* (+), and *Staphylococcus aureus* (G +), were carried out using disk diffusion method (Tuzcu et al., 2017). Bacterial suspension (0.1 ml, approximately 10^5^ CFU ml^−1^) was transferred onto a LB solid plate and coated with a sterile spreading rod. The sample films were then aseptically cut into 6‐mm‐diameter disc, sterilized by a ultraviolet lamp for 30 min and laid on the LB medium plate. The plate was then incubated at 37°C for 48 h. The diameters of the inhibition zones were measured with digital Vernier calipers in triplicate (three parallel tests):

Zoneofinhibition=Zonesfreeofbacterialgrowth–diameterofdisc.

### 2.18 Statistical analysis

The data were subjected to one‐way analysis of variance (ANOVA) and the differences between means were evaluated by Duncan's multiple comparison tests using SPSS system v.22 (SPSS Inc.). The experiments were performed with two replicates, at least.

## 3 RESULTS AND DISCUSSION

### 3.1 Characteristics of Cirish root powder

The Cirish root powder had 6.49 ± 0.27 g per 100 g moisture, 6.97 ± 0.15 g per 100 g protein, 7.08 ± 0.19 g per 100 g fat, 5.89 ± 0.12 g per 100 g ash, 73.57 ± 0.86 g per 100 g carbohydrate, and 61.35 ± 0.38 g per 100 g total fiber. The high amount of ash indicates high levels of mineral compounds. Furthermore, the Cirish root powder could be a good option for fiber enrichment. On the other hand, it is a rich source of carbohydrates.

### 3.2 Characteristics of Cirish root solution

The values of characteristics of Cirish root solution (CRS) including total carbohydrate (73.57 ± 0.86), reducing sugar (10.29 ± 0.23), extraction yield (53.19 ± 0.42), purity (33.18 ± 0.14), average chain length (5.39 ± 0.22), particle size (1089.26 ± 0.16), and zeta potential (−29.84 ± 0.36) were measured. The purity index is a quantitative indicator of extracted fructans (Pourfarzad et al., 2015). High values of this index indicate that fructans are the main component of the extract. The low purity of extracted fructans in this study can be attributed to the presence of high amounts of reducing sugars, pectinic polysaccharides, nonsugar inorganic substances, or ash‐containing nonsugar substances in the extract (Flamm et al., 2001; Karaman et al., 2011; Muhidinov et al., 2020; Pourfarzad et al., 2015).

The average chain length or degree of polymerization is considered as a qualitative indicator and shows the ability of the production process to prevent the hydrolysis of polysaccharides during extraction (Flamm et al., 2001; Pourfarzad et al., 2015). The fructans were extracted with average chain length of 5.39 ± 0.22. Therefore, the main components of CF belonged to fructo‐oligosaccharides.

### 3.3 Fourier transform infrared spectroscopy

In order to understand the changes in physical and mechanical properties of the films, monitoring the functional groups and interactions between the biopolymers through FTIR spectroscopy are crucial (De Meutter & Goormaghtigh, 2021; Payne & Veis, 1988).

**FIGURE 1:**
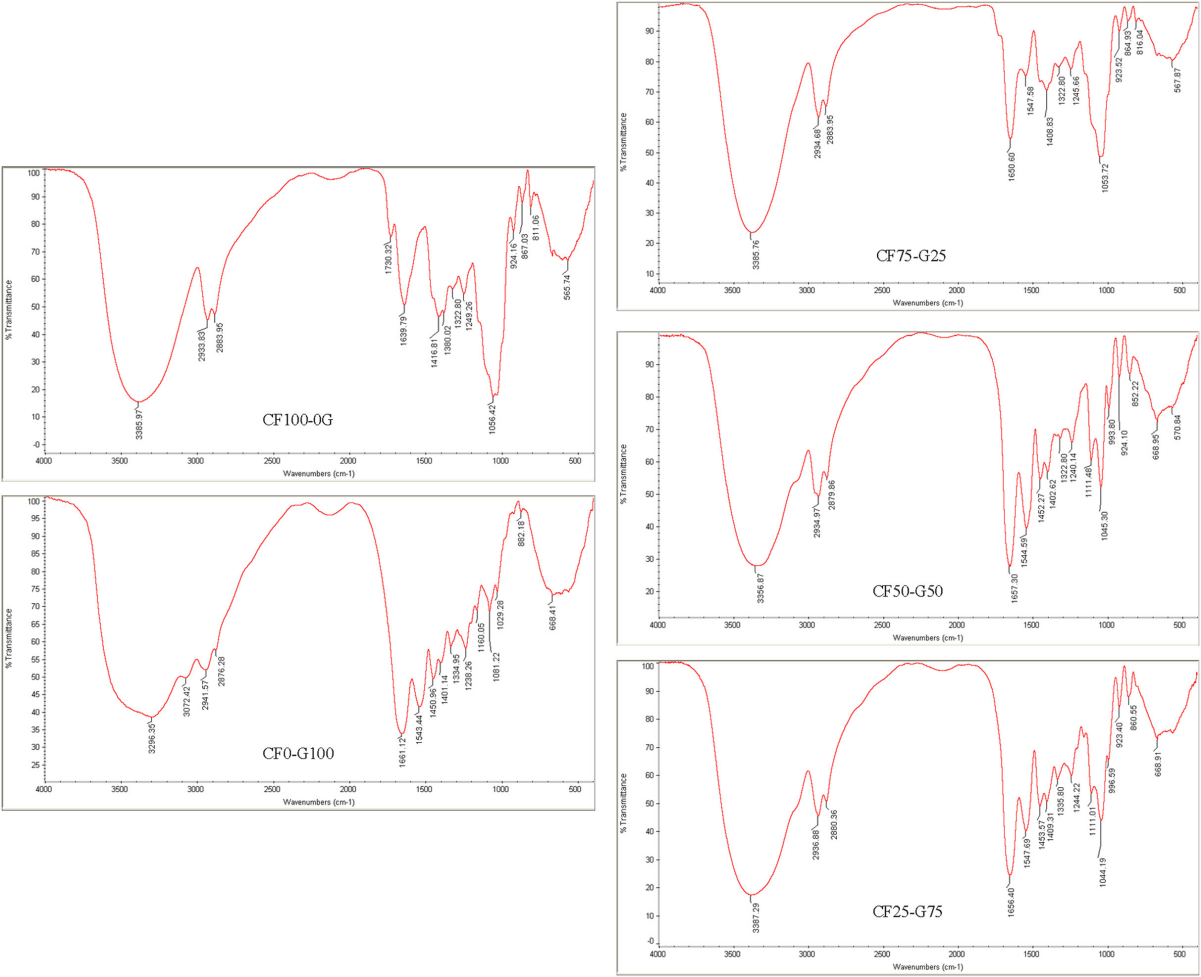
FTIR spectra of fish gelatin and Cirish fructans composite films. Cirish fructant (CF) and cold‐water fish gelatin (G) at different proportions (%) of 0:100, 50:50, 75:25, and 100:0, respectively

The spectra of gelatin film (Figure 1) showed characteristic bands at approximately 3296 cm^−1^ (amide A), 2941 cm^−1^ (amide B), 1661 cm^−1^ (amide I), 1543 cm^−1^ (amide II), and 1238 cm^−1^ (amide III). Amide A originated from the stretching and vibration of N–H and hydroxyl groups (O–H); amide B corresponds to the C–H stretching and –NH2 stretching of gelatin; amide I arises predominantly from C=O stretching vibration coupled with CN stretch, CCN deformation and in‐plane NH bending modes; amide II associated with the N–H bending and C–N stretching vibrations; amide III represented the vibration of C–N and N–H groups of bound amide as well as the wagging vibrations of CH2 groups from the glycine backbone and proline side chains (De Meutter & Goormaghtigh, 2021; Harrington & Morris, 2009; Payne & Veis, 1988). Mohajer et al. (2017), reported similar spectra for cold‐water fish skin gelatin film, where amide A, amide I, amide II, and amide III were found at the wavelengths of 3299, 1658, 1544, and 1243 cm^−1^, respectively.

The main bands associated with the CF film were appeared at FTIR spectra. The band at 3385 cm^−1^ was related to the hydroxyl group of lignin. Furthermore, the band at 1008 cm^−1^ was due to the ether bonds present in lignin. The band showed at 2933 cm^−1^ was due to the C–H stretching and bending vibrations of CH2 groups; assigned to the presence of cellulose. In addition, the peak at 1322 cm^−1^ is corresponded to cellulose. The band at 1730 cm^−1^ attributed to the C=O of the acetyl group and originated from the xylans of hemicellulose (Mohajer et al., 2017).

The peak at 1639 cm^−1^ of CF spectra indicated the presence of protein. On the other hand, the spectra of CF film showed the presence of carbohydrates. The CH–OH groups of fructo‐furanose units were appeared at 3385 cm^−1^, indicating O–H stretching vibrations, while the absorption peak of the C=O group which is part of glycosides was shown at around 1639 cm^−1^. This peak showed the presence of monomers (glucose and fructose) which means probably a part of the fructans was hydrolyzed to monomers during extraction (Zhao et al., 2011). Furthermore, the peak at 1639 cm^−^1 can be corresponding to the bound water. On the other hand, the d‐mannose was determined at 867 and 811 cm^−1^ (Figure 1).

The peaks at 924 cm^−1^ and a weak band at 800.1 cm^−1^ were due to the furanose, while the weak band at 769 cm^−1^ indicated the pyranose form of the sugars (Jahanbin et al., 2011). Furthermore, the peak at 1380 cm^−1^ indicated the angular deformation of C–H (CH3 group) in the sugar ring (Jahanbin et al., 2011). The peak appeared at 1056 cm^−1^ resulted from C–C bond and the band at 1249 cm^−1^attributed to the methyl (CH3) of the acetyl groups. On the other hand, the band detected at 1044 cm^−1^ corresponded to OH group of glycerol added as plasticizer. Therefore, the FTIR spectra proved that Cirish mainly composed of fructo‐furanose, lignin, cellulose, hemicelluloses, and monosaccharaides. Previous literatures also found the presence of heteropolysaccharides including branched arabinogalactan and linear galactomannan and glucofructan in Cirish species (Beigi & Jahanbin, 2019).

In order to study the changes in secondary structure of protein, amide I is consider the most important region (De Meutter & Goormaghtigh, 2021; Payne & Veis, 1988). It has been proven that the peaks at 1660, 1652, 1645, and 1633 cm^−1^ are related to triple‐helix, single α‐helix, random coils, and disordered structures in gelatin protein, respectively (Harrington & Morris, 2009; Payne & Veis, 1988). CF addition caused the impressive changes in gelatin secondary structure in which a frequency down‐shift from 1661 cm^−1^ in gelatin spectra toward 1650 cm^−1^ in CF75‐G25; 1657 cm^−1^ in CF50:G50 and 1656 cm^−1^ in CF25:G75 cm^−1^, suggested that the triple‐helix structure of gelatin was shifted toward α‐helix structure in composite films. It was probably due to high hydrogen bonding ability of the fructans present in the Cirish which caused interaction between functional groups of gelatin and fructans.

### 3.4 Film solubility

Films with low solubility are favorable in food packaging industry since they can resist against high humidity and delay food spoilage during storage (Chambi & Grosso, 2011). The highest solubility was observed for gelatin film with 88.15 ± 0.6% solubility (Table 1) that was in accordance with the previous studies (Hosseini et al., 2013). It was reported that gelatin film generally has high water solubility. It attributes to the –OH groups and polar peptides present in gelatin which gives it hydrophilic nature and consequently lead to more interactions with water molecules (De Meutter & Goormaghtigh, 2021; Nuanmano et al., 2015; Payne & Veis, 1988). The lowest solubility value belonged to CF film with 77.12 ± 0.3% solubility. This could be due to the presence of both soluble and insoluble fibers in Cirish solution. Therefore, the insoluble parts of the Cirish remain at the end of the solubility test. The addition of CF resulted in a significant (*p* < .05) decrease in composite films solubility. It could be ascribed to hydrogen bonds formed between gelatin and CF in the matrix, which were confirmed with FTIR spectra. In fact, covalent bonds formed between gelatin and CF decreased available hydrogen bonds to interact with water, thus led to decreasing the water solubility of composite films.

**TABLE 1:** Thickness, Moisture content, Solubility, WVP, and OP properties of fish gelatin and Cirish fructans composite films

Film type	Thickness (μm)	Moisture (%)	Solubility (%)	WVP (×10^−10^ gm^−1^ s^−1^ Pa^−1^)	OP. 10^14^ (cm^3^ m^−1^ s^−1^ Pa^−1^)
CF0‐G100	69.38 ± 0.15^e^	16.05 ± 0.11^d^	88.15 ± 0.42^a^	2.86 ± 0.21^a^	3.26 ± 0.08^a^
CF25‐G75	71.26 ± 0.21^d^	18 ± 0.19^c^	84.55 ± 0.23^b^	2.35 ± 0.24^ab^	2.84 ± 0.12^ab^
CF50‐G50	75.09 ± 0.17^c^	24 ± 0.22^b^	80.52 ± 0.27^c^	2.18 ± 0.22^bc^	2.40 ± 0.05^bc^
CF75‐G25	78.16 ± 0.09^b^	25 ± 0.26^b^	78.42 ± 0.12^d^	1.93 ± 0.18^cd^	1.98 ± 0.06^cd^
CF100‐G0	86.45 ± 0.13^a^	27.8 ± 0.16^a^	77.12 ± 0.31^d^	1.89 ± 0.21^d^	1.53 ± 0.05^d^

*Note*: Reported values correspond to the mean ± standard deviation. Different letters in the same column indicate significant differences (*p* < .05). Cirish fructans (CF) and cold‐water fish gelatin (G) at different proportions (%) of 0:100, 50:50, 75:25, and 100:0, respectively.

Abbreviations: OP, oxygen permeability; WVP, water vapor permeability.

### 3.5 Film thickness

Film thickness is a very important parameter which might be correlated with the mechanical, permeability, and resistance to water properties of the films (Chambi & Grosso, 2011; Hanani et al., 2012). The thickness of films was varied from 69.38 ± 0.15 to 86.45 ± 0.13 μm (Table 1). The results showed that gelatin film was thinner than CF film. The thickness of composite films was increased when CF/gelatin ratio was increased. It might be related to greater particle size, impurities, and lower solubility of CF.

### 3.6 Moisture content

The moisture content of all the films is shown in Table 1. The moisture content of CF film was 27.8% moisture, which was greater than gelatin film with 16.05% moisture content. The results revealed that incorporation of CF increased the moisture content of composite films which CF75‐G25 film had highest moisture content. These findings might be related to high fructans, reducing sugar, and monosaccharaides content of CRS and their hygroscopic characteristics (Flamm et al., 2001; Muhidinov et al., 2020; Pourfarzad et al., 2015).

### 3.7 Morphological characteristics

The microstructure of surface and cross‐section of the films depends on the interactions between film components and drying conditions. The study of these properties can help us to elucidate the structure characteristics, such as barrier and mechanical properties (Hanani et al., 2012).

**FIGURE 2:**
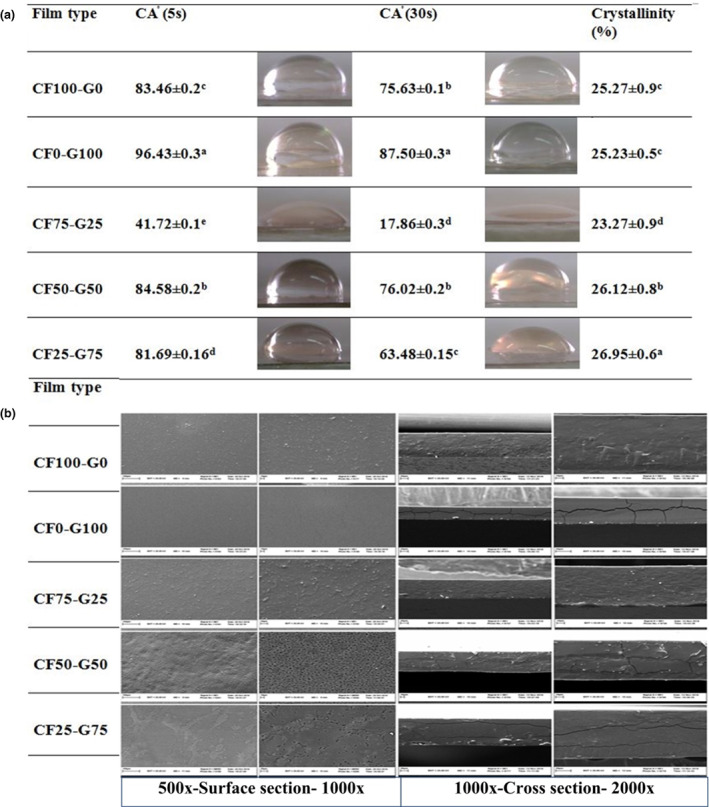
(a) Surface hydrophobicity (contact angle) properties and crystallinity of fish gelatin and Cirish fructans composite films. Reported values correspond to the mean ± standard deviation. Different letters in the same column indicate significant differences (*p* < .05). (b) Microstructure of surface and cross‐section of fish gelatin and Cirish fructans composite films. Cirish fructant (CF) and cold‐water fish gelatin (G) at different proportions (%) of 100:0, 0:100, 75:25, 50:50, and 25:75, respectively

The surface and cross‐sectional micrographs are shown in Figure 2. The micrographs of CF film exhibited smooth and uniform surface, where polysaccharide chains oriented to form a continuous and dense network. It was revealed that cross‐section of CF film was compact, indicating its good film‐forming capacity. However, gelatin film showed obvious cracks on the surface and phase separation was found on cross‐section. It showed that gelatin had brittle structure which fractured when liquid nitrogen was used for preparation of film for SEM test. Accordingly, Gómez‐Guillén et al. (2007) and Pranoto et al. (2007) observed that fish gelatin film had fracture surface. Incorporation of 25% CF to gelatin resulted in a rough surface with a few pores and uneven cross‐sections with cracks. However, increasing the CF content enhanced characteristics of composite films. The uniform surface without spots and cracks were observed for CF75‐G25 composite film and no phase separation was found as can be observed in both surface and cross‐sectional images. It implies the formation of a cross‐linking network structure between gelatin, CF, and phenolic components.

It seems that the viscosity of CF solution is lower than gelatin. Therefore, lower viscosity in CF75‐G25 and CF50‐G50 solutions might cause higher mobility in the polymer molecules. Thereby, CF molecules could distribute in gelatin matrix more homogenously and create more uniform network. The results demonstrated that CF and gelatin seem to be compatible to form film due to their similar hydrophilic character. The obtained results supported the low WVP and good mechanical properties of composite films with high CF content.

### 3.8 Water vapor permeability

Edible films with low WVP are preferred to retard the moisture transfer between foods and their surrounding environment (Chambi & Grosso, 2011). The WVP values of CF, gelatin, and composite films are shown in Table 1. The WVP of the pure gelatin film was 2.86 × 10^−10^ (gm^−1^ s^−1^ Pa^−1^). However, CF film had lower WVP (1.89 × 10^−10^ (gm^−1^ s^−1^ Pa^−1^)). The WVP of composite films ranged from 1.93 to 2.35 × 10^−10^ (gm^−1^ s^−1^ Pa^−1^), in which lower WVP was obtained when high level of CF was incorporated (CF75‐G25). Consequently, this will prevent food spoilage. The similar results were observed by Hosseini et al. (2013) who found that the protein–polysaccharide composite films had low WVP when comparing with gelatin film. In this regard, Giménez et al. (2013) and Pranoto et al. (2007) concluded that polysaccharides might enhance the cross‐linking of protein and decrease the free volume of the polymeric matrix, and thus resulting in lower WVP.

These results were supported by SEM observations as discussed previously. The denser and uniform polymeric matrix was formed resulting from the interactions between gelatin, polyphenols, and CF, which impedes passing of water molecules through the context. In addition, the existence of cracks and pores in the gelatin film and composite films with high level of gelatin (CF50‐G50 and CF25‐G75) could be a reason for their high WVP. The presence of phenolic compounds in Cirish solution also can influence in decreasing WVP. In fact, the interactions of phenolic compounds with hydrophobic regions of gelatin could limit the penetration of water molecules (Wu et al., 2013). Similarly, Giménez et al. (2013) reported that the WVP of agar–gelatin films were decreased when green tea extract was incorporated. In fact, the incorporation of CF helped to maintain the composite film integrity when immersed in water.

### 3.9 Oxygen permeability

Biocomposite films possess excellent oxygen barrier properties because of their considerable hydrogen bonds, high chains density, and semi‐crystalline state. These features make them suitable choice for food packaging to reduce their respiration rate and retard spoilage (Yadav et al., 2020).

The OP of gelatin film (3.26 ± 0.11) was significantly higher than CF film (1.53 ± 0.12; Table 1). Indeed, the OP decreased when the CF content rose. The OP strongly depends on the structure of films and the interaction between the polymer matrix and O_2_. On the other hand, the promotion of the oxygen barrier in composite films with greater CF content could be related to their high crystallinity, since crystallinity increases the ability of the matrix against oxygen transfer. It was previously reported that the CF have semi‐crystalline nature (Pourfarzad et al., 2015). In this regard, Wang et al. (2017) found that incorporation of konjac Glucomannan (KGM) improved OP of KGM–zein composite film.

### 3.10 Surface hydrophobicity properties (Contact angle)

Surface hydrophobicity is very important for food packaging materials. Contact angle is used to measure the hydrophobicity/hydrophilicity of film surface (Wang et al., 2017). Generally, the film surface is considered hydrophobic when the contact angle is large (θ > 90°), while the film with small angle (θ < 90°) implies a hydrophilic surface (Dammak et al., 2017). The contact angle at initial and after 30 s of connection of water droplets with the film surface is reported in Figure 2. It was observed that the contact angle of all samples was reduced after 30 s. The contact angle of gelatin film was 83.46 ± 0.2°, while CF film had lower contact angle (81.69°), implying that CF film had more hydrophilic surface resulting from more hydrophilic groups, such as carboxyl and hydroxyl present in fructans.

Generally, the incorporation of CF in composite films improved the surface hydrophobicity. This is probably because CF and gelatin could interact with each other by hydrogen bonds, resulting in burring active sites inside the film surface and improving the hydrophobic character. In this regard, Tao et al. (2018) also concluded that the cross‐linking between gelatin and starch by hydrogen bonds was a major factor to improve the hydrophobicity of composite film (Tao et al., 2018). However, the interesting finding was that the composite film with equal amount of CF and gelatin (CF50‐G50) had the lowest contact angle. It might be due to the high amount of pores on the surface of composite film which was observed at SEM micrographs.

### 3.11 Mechanical properties

The mechanical properties, such as the tensile strength (TS) and elongation at break (EAB), are closely related to the distribution and density of the intra‐ and intermolecular interactions between polymer chains, arrangement of different components in the matrix, and cohesion forces in the film matrix. These features reflect the ability of the film to withstand the external stresses inserted during their transportation (Wang et al., 2017; Yadav et al., 2020).

The mechanical properties of the films are presented in Table 2. The TS of the gelatin film was 7.04 MPa which was similar to the results obtained by Hanani et al. (2012) and Hosseini et al. (2013) who reported 7.22 and 7.44 MPa for fish gelatin films, respectively. The CF film had the highest TS, while the lowest TS belonged to CF25‐G75 composite film with 17.89 and 5.73 MPa TS, respectively. Previous studies have revealed that protein films are generally brittle and susceptible to cracking, thus have low TS as a result of strong cohesive energy density of the polymer (Chambi & Grosso, 2011; Hanani et al., 2012; Hosseini et al., 2013). This finding was confirmed by SEM images where non‐continues network along with cracks were observed for CF25‐G75 composite film, implying that the CF and G were not compatible at this ratio. Addition of 50% and 75% CF caused a significant (*p* < .05) increase in the TS value which were probably due to the increasing of continuity in the gelatin matrix with CF addition. Chambi and Grosso (2011) have reported that the intermolecular interactions between the polymers are the main cause for increasing the TS. It was shown that polysaccharides could interweave with gelatin chains, thus increase the TS (Chambi & Grosso, 2011; Pranoto et al., 2007). The presence of OH groups at the polymer side chains of carbohydrate and/or proteins could contribute at formation of intermolecular hydrogen bonds between polymers and also between polymers and phenolic compounds present in the Cirish solution, improving the film strength.

**TABLE 2:** The mechanical and thermal properties of fish gelatin and Cirish fructans composite films

Film type	TS (MPa)	EAB (%)	Young's modulus (MPa)
CF0‐G100	7.04 ± 0.21^c^	1.66 ± 0.05^a^	88.15 ± 0.46^a^
CF25‐G75	5.73 ± 0.09^d^	0.83 ± 0.02^c^	84.55 ± 0.23^b^
CF50‐G50	10.27 ± 0.15^b^	1.02 ± 0.03^bc^	80.52 ± 0.27^c^
CF75‐G25	16.67 ± 0.18^ab^	1.66 ± 0.02^a^	78.42 ± 0.32^d^
CF100‐G0	17.89 ± 0.11^a^	1.46 ± 0.06^ab^	77.12 ± 0.31^e^

*Note*: Reported values correspond to the mean ± standard deviation. Different letters in the same column indicate significant differences (*p* < .05). Cirish fructant (CF) and cold‐water fish gelatin (G) at different proportions (%) of 0:100, 50:50, 75:25, and 100:0, respectively.

Abbreviations: EAB, elongation at break; TS, tensile strength.

The EAB is a measure of the stretchability of a film, and shows its ability to deform under pressure (Hanani et al., 2012). Low values of EAB imply that the film is brittle (Chambi & Grosso, 2011). The EAB of the gelatin film was 1.66%, while the CF had 1.46% EAB. The results indicated that incorporation of CF decreased the EAB of composite films. A similar behavior was observed by Hosseini et al. (2013) who showed that the addition of chitosan to composite films manufactured from gelatin–chitosan increased TS and decreased EAB. It was observed that the increasing CF content resulted in high EAB, suggesting that the viscous texture of CRS contributes to the stretchable gelatin film network. The highest EAB belonged to CF75‐G25 which confirmed by SEM and cross‐sectional images where continuous uniform surface without cracks and phase separation were found. As mentioned, high ratio of CF to gelatin caused more cohesive and flexible matrixes, resulting in high EAB. These results were also supported by Young's modulus (YM). The YM was increased when CF content was increased. YM is a measure of the intrinsic stiffness of films (Chambi & Grosso, 2011; Hanani et al., 2012; Hosseini et al., 2013). Therefore, incorporation of CF increased the stiffness of composites films. The present study showed that CF formed the similar flexible/stretchable film to gelatin while it had more intrinsic stiffness with higher TS. On the other hand, the results indicated that the CF showed compatibility with gelatin matrix to manufacture superior film.

### 3.12 Thermal properties

**FIGURE 3:**
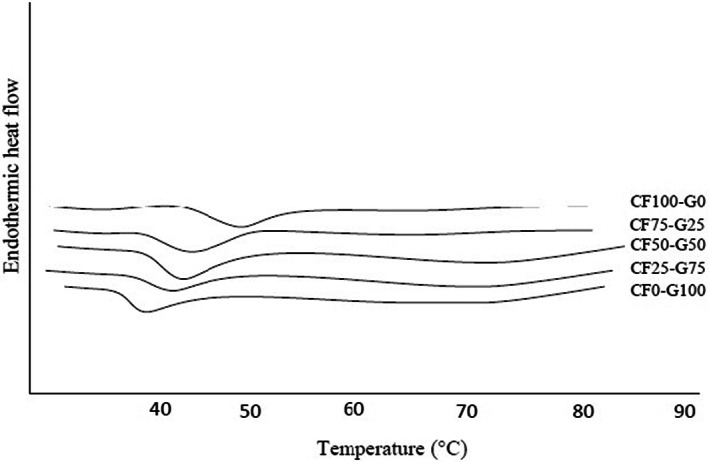
DSC thermogram of fish gelatin and Cirish fructans composite films. Cirish fructant (CF) and cold‐water fish gelatin (G) at different proportions (%) of 0:100, 50:50, 75:25, and 100:0, respectively

The glass transition is associated with molecular segmental motion of disordered structure (amorphous phase). It indicates the temperature that disrupts the polymer interactions formed during film preparation (Dai et al., 2006; Wang et al., 2017). The glass transition of proteins is associated with the melting of crystalline domains and α‐amino acid blocks present in the polypeptide chains (Dai et al., 2006; Hosseini et al., 2013). The Tg values are illustrated in Figure 3.

The gelatin had Tg of 38.83 ± 0.15°C which was lower than those of CF film (47.4 ± 0.17°C). The Tg of composite films were increased gradually from 42.04 ± 0.15 to 43.29 ± 0.11°C when CF content was increased. It was revealed that the addition of CF more likely facilitated the intermolecular interactions between protein and polysaccharide in the film matrix, thereby reduced the mobility of gelatin chain and increased thermal stability, thus resulting in high Tg. In this regard, it has been reported that the promoting chain rigidity and the intensity of both inter‐ and intramolecular interactions could cause increase in the Tg and transition enthalpy as a result of hindrance of internal rotation along the macromolecular chains (Dai et al., 2006; Hosseini et al., 2013; Wang et al., 2017). Furthermore, it had been concluded that the reduction in the number of hydrogen bonds with a simultaneous increase in the extent of covalent cross‐linking cause the increase in thermal stability (Giménez et al., 2013; Hosseini et al., 2013; Wang et al., 2017). Pranoto et al. (2007) supported our claim and reported that protein–polysaccharide interactions could lower the mobility of protein chain and increase Tg values (Pranoto et al., 2007). On the other hand, Soo and Sarbon (2018) reported that cross‐linking reaction between gelatin matrix and rice flour components reduced the mobility of biopolymer chains, thus produced highly heat‐stable films. The SEM and mechanical properties were confirmed the thermal behavior of the samples in which the structural integrity of gelatin‐based network was improved when CF was presented, ascribed to high TS and low EAB.

### 3.13 Total phenolic content and antioxidant activity

**FIGURE 4:**
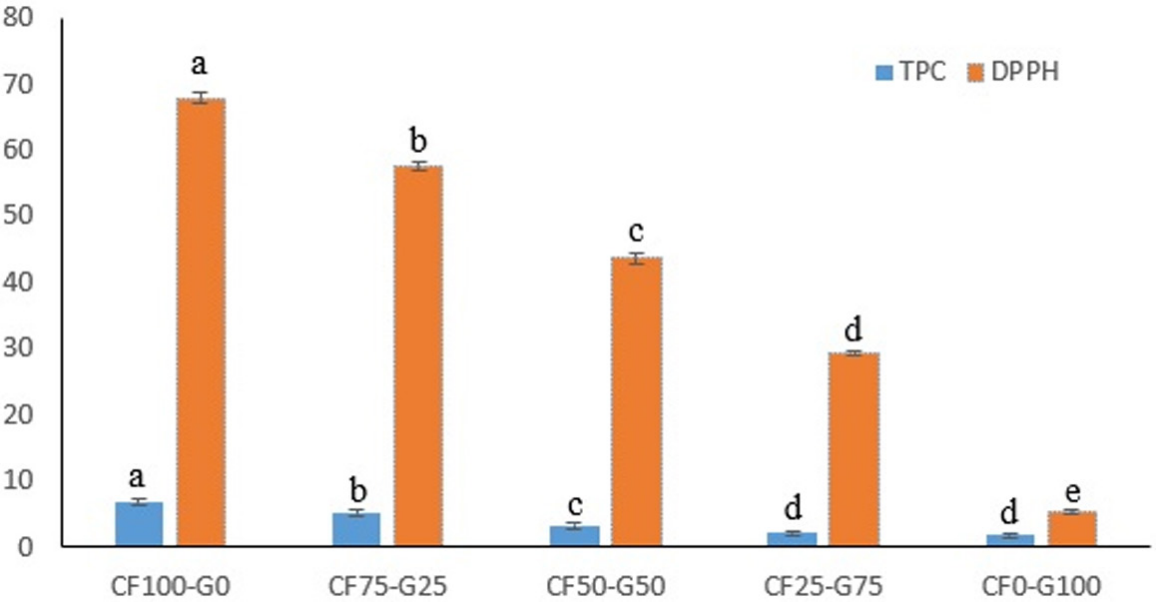
Total phenolic content (TPC) and antioxidant activity (DPPH) of fish gelatin and Cirish fructans composite films. Cirish fructant (CF) and cold‐water fish gelatin (G) at different proportions (%) of 0:100, 50:50, 75:25, and 100:0, respectively. TPC, total phenolic content

Phenolic compounds and their secondary metabolites are active components in a plant that may be able to inhibit oxidation by acting as hydrogen donors, reducing agents, singlet oxygen quenchers, and metal chelator (Gómez‐Estaca et al., 2009; Gómez‐Guillén et al., 2007; Tuzcu et al., 2017). The results indicated that the TPC of the CF film was 6.73 mg Gallic acid g^−1^, which was higher than gelatin film (1.89 mg Gallic acid g^−1^; Figure 4). Incorporation of CF resulted in composite films with high phenolic content. The obtained results were in accordance with Karaman et al. (2011) who reported that CRS extracted by methanol, ethanol, and aqueous media had total phenolic content at the range of 7.15–31.92 (mg GAE g^−1^) and antioxidant activity at the range of 21.01–81.72 (mg AAE g^−1^).

DPPH (DPPH; C18H12N5O6, M = 394.33) consists of a nitrogen free radical and a proton radical scavenger and shows the potential of antioxidants to inhibit free radicals and generating nonradicals form DPPH. This feature attributes to hydrogen‐donating ability of the compounds (Gómez‐Estaca et al., 2009; Kedare & Singh, 2011).

The results suggested that the CF film possesses strong scavenging activity against DPPH radical (67.86%; Figure 4). Adding CF to gelatin film promoted antioxidant activity from 5.26% for gelatin film to 57.53% for CF75‐G25 film, suggesting that CF‐based films could be used as active films that can delay or inhibit oxidation. The inherent antioxidant activity of gelatin film might have originated from the peptide fractions and particular amino acids such as proline, hydroxyproline, and glycine which contribute to electron donation (López et al., 2017). A study conducted by Gómez‐Guillén et al. (2007) revealed that films formed from tuna‐fish gelatin exhibited antioxidant activity. The oxidation of phenolic compounds of CF to radicals can cause covalent interactions with the amino acids of gelatin which contribute to the antioxidant activity of film (Gómez‐Estaca et al., 2009; Gómez‐Guillén et al., 2007; Tuzcu et al., 2017).

The antioxidant activity of CF film is attributed to the presence of bioactive compounds such as polyphenol, essential oils, and water‐soluble compounds. Kanaani and Mohamadi Sani (2015) have shown that Cirish root is a source of essential oils; n‐Octane and n‐Decane are the major constituents which contribute at antioxidant activity (Kanaani & Mohamadi Sani, 2015). Previous studies have shown that the major volatile components of Cirish root are Carvone (terpenoid), carvacrol (monoterpenoid phenolic compound), pentane, 2‐methyl‐ (E) caryophyllene (natural bicyclic sesquiterpene), valencene, cis‐calamenene, cadalene, and acetic acid, contributing to the antioxidant and antibacterial activity (Karaman et al., 2011) which are present in the root as a defense mechanism toward insects, fungi, and other environmental stress. On the other hand, glucomannans are bioactive water‐soluble polysaccharides present in Cirish root, contributing to the antioxidant activity (Muhidinov et al., 2020).

### 3.14 Antimicrobial activity

The antimicrobial activity of films against gram‐positive (*B*. *subtilis*, *S. aureus*, and *L. monocytes*) and gram‐negative (*E. coli*) microorganisms is presented in Table 3. The gelatin film did not show antimicrobial activity while the CF film inhibited the growth of all microorganisms. The results showed that incorporation of CF gave antimicrobial characteristic to composite films. It was revealed that there was no difference in antimicrobial activity of CF between gram‐positive bacteria and gram‐negative bacteria*. E. coli* (−) were the most sensitive with inhibition diameters of 19.45 mm while *S. aureus* (+) were the most resistant with inhibition diameters of 4.26 mm.

**TABLE 3:** Antimicrobial activity (inhibition zone) of fish gelatin and Cirish fructans composite films

Film type	Inhibition diameters (mm)			
	*E. coli* (−)	*B. subtilis* (+)	*L. monocytes* (+)	*S. aureus* (+)
CF0‐G100	0.00 ± 0.00^e^	0.00 ± 0.00^e^	0.00 ± 0.00^d^	0.00 ± 0.00^d^
CF25‐G75	3.26 ± 0.13^d^	2.12 ± 0.15^d^	0.00 ± 0.00^d^	0.00 ± 0.00^d^
CF50‐G50	9.09 ± 0.11^c^	8.13 ± 0.07^c^	1.26 ± 0.08^c^	0.88 ± 0.03^c^
CF75‐G25	16.16 ± 0.09^b^	12.06 ± 0.10^b^	4.36 ± 0.21^b^	1.36 ± 0.07^b^
CF100‐G0	19.45 ± 0.20^a^	18.15 ± 0.16^a^	7.26 ± 0.17^a^	4.26 ± 0.14^a^

*Note*: Reported values correspond to the mean ± standard deviation. Different letters in the same column indicate significant differences (*p* < .05). Cirish fructant (CF) and cold‐water fish gelatin (G) at different proportions (%) of 0:100, 50:50, 75:25, and 100:0, respectively. *Escherichia coli* (G −), *Bacillus subtilis* (G +), and *Staphylococcus aureus* (G +).

Generally, it is believed that gram‐negative organisms are less sensitive toward antimicrobial components because of outer lipopolysaccharides membrane around their cell wall that provide a surface hydrophilicity, thus preventing the access of antimicrobial components with mostly hydrophobic nature (Tuzcu et al., 2017). However, there are studies with opposite results. Our results were in agreement with the study of Tuzcu et al. (2017) who concluded that CRS had antimicrobial activity against both of gram‐negative (*E. coli*) and gram‐positive (*B. subtilis*) organisms.

The antimicrobial activity of CRS could be attributed to phenolic compounds, essential oils, and volatile components. It has been reported that n‐Octane and n‐Decane as main components of Cirish essential oils seem to be responsible for antimicrobial activity due to their hydrophobic nature (Kanaani & Mohamadi Sani, 2015). Kanaani and Mohamadi Sani (2015) revealed that the essential oils of aerial parts of Sonchus arvensis and roots of *Eremurus spectabilis* (Cirish) could inhibit the growth of gram‐positive and gram‐negative bacteria. On the other hand, volatile components of CRS could contribute to antibacterial activity (Karaman et al., 2011).

## 4 CONCLUSION

The current research indicated that the active films were successfully developed through incorporation of Cirish fructans (CF) to cold‐water fish gelatin, reflecting the antioxidant and antimicrobial potential of Cirish. It was found that adding CF improved physical, functional, mechanical, and barrier properties of gelatin film. CF incorporation formed harder films with higher resistance to break and more extensibility. The formation of denser and uniform polymeric matrix impedes passing of water and oxygen molecules, thereby reducing WVP and OP. The increasing Tg of composite films with CF incorporating confirmed improving structural integrity of the film's matrix. The morphological observation demonstrated that incorporation CF led to uniform surface without spots, cracks, and phase separation, implying that CF and gelatin were compatible to form film. The desirable antioxidant properties, DPPH scavenging activity, and total phenolic content were achieved when CF was added. Moreover, the antimicrobial activity toward both of gram‐positive bacteria and gram‐negative bacteria was identified. This study revealed high potential of CF and composite CF‐G films to be used as active packaging in commercial food systems to retard spoilage and extend shelf life.
